# Supply chains in health emergency response

**DOI:** 10.2471/BLT.21.021221

**Published:** 2021-12-01

## Abstract

Increasing health emergencies are driving unprecedented demand for effective supply chains to support emergency response. Andréia Azevedo Soares reports.

“Around 20 000 health workers have not received their salary in four months, we’re running out of essential medicines as well as medical and hygiene consumables and fuel for our ambulances is running low.”

For Dr Mohammad Dawood Ayoubi, Coordinator of health programmes and public clinics in Kandahar, Afghanistan, these are the signs of a health system in distress.

The system in question is supported by Sehatmandi, a project that supports delivery of Afghanistan’s Basic Package of Health Services and Essential Package of Hospital Services across the country’s 34 provinces. Or at least that is what it was doing until August, when the Taliban seized control of the country, and the multiple donors on which the project depends froze their contributions.

“The funding freeze put 90% of the 2309 health facilities in the system at risk,” explains Ayoubi, who works for the nongovernmental organization BARAN & OHPM (Bu Ali Rehabilitation and Aid Network and Organization for Health Promotion and Management), one of the nongovernmental organizations contracted to deliver Sehatmandi services.

The World Health Organization (WHO) was one of several organizations to respond, shipping urgently needed supplies shortly after the country came under Taliban control.

“The first shipment arrived in Mazar-i-Sharif Airport, in the north of Afghanistan, on 30 August,” says Dr Richard Brennan, emergency director for WHO’s regional office for the Eastern Mediterranean. As of 3 November, WHO had delivered a total of 228 million metric tonnes of supplies, including essential medicines such as insulin, medical consumables, trauma and surgery kits, and coronavirus disease 2019 (COVID-19) testing kits.

According to Brennan, the health emergency in Afghanistan is just one among many unfolding in the region. “We are currently responding to 10 large-scale humanitarian crises, including five of the seven largest in the world, involving around 43% of the 244 million people estimated to need humanitarian assistance,” he says.

WHO’s activities in emergency response have expanded considerably since the creation of the Organization’s Health Emergencies Programme in 2016. That programme was set up in the aftermath of the 2014–2016 Ebola outbreaks in West Africa and since 2016 has been instrumental in responding to multiple events including cholera, yellow fever and malaria outbreaks in Africa, the 2020 explosion in Lebanon and ongoing humanitarian emergencies in countries such as Afghanistan, Ethiopia, Iraq, Libya, Myanmar, Nigeria, the occupied Palestinian territory, Somalia, South Sudan, Syria and Yemen.

“We are currently responding to 10 large-scale humanitarian crises.”

Richard Brennan

Underpinning all WHO’s health emergencies work are Operations Support and Logistics (OSL) activities, designed to back up health and social service systems unable to meet the health needs of vulnerable populations. These range from end-to-end supply chain and specialized health logistics, which can include biological sample transportation and dead body management, to field capacity support such as accommodation and office space, and information and communications technology.

At the core of WHO’s OSL work is a logistics hub located to the west of Maktoum International Airport in Dubai in the United Arab Emirates. Set up in 2015, the hub was established in Dubai partly to take advantage of the city’s unique connectivity. “We are within 4–8 hours’ flight of roughly two thirds of the global population,” says Robert Blanchard, WHO’s Dubai-based team leader for emergency operations.

Dubai also offered considerable capacity for expansion in the complex of warehouses founded and managed by the International Humanitarian City, the largest and only non-profit, independent humanitarian free-zone authority in the world. The City hosts a community of 80 members comprised of United Nations (UN) agencies, and governmental and nongovernmental organizations.

The City’s capacity to stretch has proven invaluable as WHO’s emergency work has grown, notably in response to the COVID-19 pandemic. “The combined UN and partner response to COVID-19 has placed Dubai at the physical centre of a transportation and hub network that covers the globe,” says Paul Molinaro, head of OSL in the WHO Health Emergencies Programme.

In 2019, the hub’s footprint expanded from 4000 square metres to more than 12 000, mainly in response to the humanitarian emergency in Yemen. In 2020, the hub jumped to 20 000 square metres, largely in response to the COVID-19 pandemic which drove a massive spike in demand for personal protective equipment, laboratory reagents and biomedical equipment.

“The Dubai hub has been a game changer during the pandemic,” says Brennan. “So far it has supported 120 countries, across the six WHO regions through more than 700 shipments. That is more than the previous five years combined.”

And the pace is only accelerating. In the first week of September, the Dubai hub dispatched 450 metric tonnes of medical supplies, four times its typical weekly average, in support of the cholera outbreak response in Nigeria, critical shortages of medicines in Afghanistan, and trauma and surgical supplies to Syria and Yemen.

In the same week, 85 metric tonnes of medical supplies were delivered to Ethiopia, the largest single shipment of humanitarian cargo airlifted by the hub to date. In October, the hub began delivering 283 metric tonnes of critical medicines and health supplies to Khartoum, Sudan through an air bridge supported by His Highness Sheikh Mohammed bin Rashid Al Maktoum, Vice-President and Prime Minister of the United Arab Emirates. The first delivery of supplies arrived on 24 October and was distributed to health facilities in 11 states across Sudan.

As impressive as all this is, Molinaro is at pains to emphasize the extent to which WHO works alongside other international organizations and governmental and nongovernmental organizations to meet its deadlines. “Collaboration is key,” he says. “For example, in regard to our Afghanistan response, we have been able to plug into the World Food Programme aviation and warehouse network, utilize global freight agreements established by the United Nations Children’s Fund (UNICEF), leverage airlines’ cargo capacity from Qatar and Pakistan, including utilizing donated cargo space, and optimize loads by sharing availability with other response partners, including UNICEF, the UNFPA (United Nations Population Fund) and the IFRC (International Federation of Red Cross and Red Crescent Societies).”

“Dubai [is] at the physical centre of a transportation and hub network that covers the globe.”

Paul Molinaro

While gratified to see WHO’s Dubai operation expanding to meet rapidly growing need, Blanchard highlights challenges that include fragmented systems for the management of supplies as well as the supply chain constraints impacting many of the countries WHO serves and operates in.

He is hopeful that the situation will improve as the Organization embarks on establishing an end-to-end supply chain management system as part of its ongoing transformation, an initiative designed to increase its impact at country level and make it fit-for-purpose in the era of the sustainable development goals.

“Supply chain management systems are crucial to ensuring timely delivery which, in many cases, is a matter of life and death,” Blanchard says. “Our biggest enemy is time. Not just because many of our products have limited shelf lives but because the people waiting at the other end sometimes need them to get through another day.”

Blanchard also points out that optimized supply chain management systems can generate the data required by managers and clinicians implementing health emergency response on the ground. “This is exactly what the doctors and emergency managers in the field as well as senior managers within the Organization are calling for,” he says. “They need to be able to see what inventory levels are, where these inventories are held, how long it will take to reach them and what will be required in terms of cost and time to deliver them to where they need to go.”

Needless to say, the ultimate beneficiaries of optimized supply chain management are the people who find themselves bereft of supplies and services, in countries such as Afghanistan.

As of mid-November, health facilities in all 34 Afghan provinces, including those coordinated by Dr Ayoubi, were eagerly awaiting the arrival of the next cargo of health supplies and the money needed to pay health worker salaries. Despite reports of money getting through, Ayoubi says cash is in short supply and has been for some time. “One of our core funders stopped paying contracted NGOs, including ourselves, for delivering the basic health package in April 2021,” he says. “Nor have they paid the staff of the health centres. Health professionals have continued working so far, but the situation is very challenging."

Meanwhile, there is growing concern about outbreaks of measles and acute diarrhoea, about food insecurity, drought and the onset of winter. Tackling those challenges will require a massive multi-agency, multi-stakeholder response.

“The necessity of an agile, well-funded and coordinated logistics and supply operation, embedded as a core pillar within the leadership of the overall global response, has never been clearer,” says WHO’s Molinaro. “Our experience supporting the global emergency response from Dubai over the last five years has only reinforced this lesson.”

**Figure:**
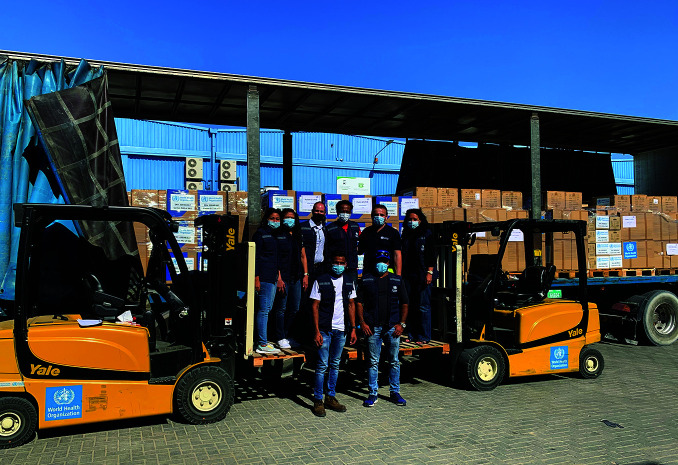
WHO’s Dubai hub team.

Courtesy of Robert Blanchard

**Figure:**
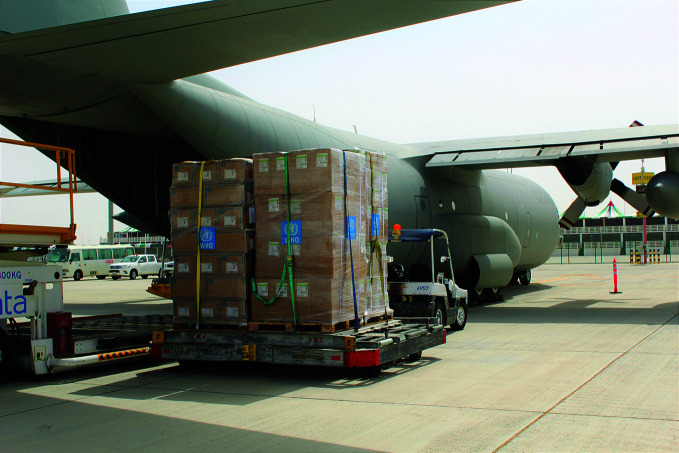
Emergency supplies destined for Somalia being loaded into a military transport.

Courtesy of Robert Blanchard

